# Computational Modeling of Contrast Sensitivity and Orientation Tuning in First-Episode and Chronic Schizophrenia

**DOI:** 10.1162/CPSY_a_00005

**Published:** 2017-12-01

**Authors:** Steven M. Silverstein, Docia L. Demmin, James A. Bednar

**Affiliations:** 1 Rutgers University Behavioral Health Care, Piscataway, New Jersey, USA; 2 Robert Wood Johnson Medical School Department of Psychiatry, Rutgers University, Piscataway, New Jersey, USA; 3 Department of Psychology, Rutgers University, Piscataway, New Jersey, USA; 4 School of Informatics, University of Edinburgh, Edinburgh, Scotland

**Keywords:** schizophrenia, vision, perception, contrast sensitivity, orientation tuning, modeling, first episode, Hebbian learning, gain control, inhibition, excitation

## Abstract

Computational modeling is a useful method for generating hypotheses about the contributions of impaired neurobiological mechanisms, and their interactions, to psychopathology. Modeling is being increasingly used to further our understanding of schizophrenia, but to date, it has not been applied to questions regarding the common perceptual disturbances in the disorder. In this article, we model aspects of low-level visual processing and demonstrate how this can lead to testable hypotheses about both the nature of visual abnormalities in schizophrenia and the relationships between the mechanisms underlying these disturbances and psychotic symptoms. Using a model that incorporates retinal, lateral geniculate nucleus (LGN), and V1 activity, as well as gain control in the LGN, homeostatic adaptation in V1, lateral excitation and inhibition in V1, and self-organization of synaptic weights based on Hebbian learning and divisive normalization, we show that (a) prior data indicating *increased* contrast sensitivity for low-spatial-frequency stimuli in first-episode schizophrenia can be successfully modeled as a function of reduced retinal and LGN efferent activity, leading to overamplification at the cortical level, and (b) prior data on *reduced* contrast sensitivity *and* broadened orientation tuning in chronic schizophrenia can be successfully modeled by a combination of reduced V1 lateral inhibition and an increase in the Hebbian learning rate at V1 synapses for LGN input. These models are consistent with many current findings, and they predict several relationships that have not yet been demonstrated. They also have implications for understanding changes in brain and visual function from the first psychotic episode to the chronic stage of illness.

## INTRODUCTION

Schizophrenia is a disabling psychiatric disorder that is characterized by perceptual distortions, hallucinations, delusions, disorganized thinking and speech, bizarre behavior, loss of motivation, motor abnormalities, and a decline in multiple aspects of functioning. However, despite a massive accumulation of data in recent decades regarding schizophrenia and its treatment, its etiology and pathophysiology remain unclear. A major reason for this lack of progress is the absence of a cohesive theoretical framework within which to understand the wide range of research findings. Consequently, it has been proposed that computational modeling efforts can assist in clarifying the core biobehavioral processes inherent to the disorder (Silverstein, Moghaddam, & Wykes, 2014). Specifically, modeling can help (a) reconcile different types of empirical findings, including those from different levels of functioning (e.g., neural circuits, cognition, symptoms), and (b) identify hypotheses that seem most (and least) likely to be con firmed in future animal and human studies, thereby potentially saving time and resources and hastening novel treatment development. A strength of some current computational models is that they can account for both cognitive and clinical aspects of schizophrenia (Adams, Huys, & Roiser, 2016; Adams, Stephan, Brown, Frith, & Friston, 2013; Clark, 2013; Friston, Stephan, Montague, & Dolan, 2014; Montague, Dolan, Friston, & Dayan, 2012; X. J. Wang & Krystal, 2014). To our knowledge, however, no prior model has attempted to account for the common perceptual disturbances in the disorder. Therefore, in this article, we model aspects of low-level visual processing and demonstrate how this can lead to testable hypotheses about both the neural mechanisms involved in visual abnormalities in schizophrenia and the relationships between these mechanisms and psychotic symptoms.

Multiple aspects of visual processing are impaired in schizophrenia, including visual acuity (Smith, Pantelis, McGrath, Tangas, & Copolov, 1997), peripheral vision (Kraehenmann, Vollenweider, Seifritz, & Kometer, 2012), stereopsis (Schechter et al., 2006), contrast sensitivity (CS; Calderone et al., 2013), spatial frequency (SF) processing (Shoshina, Shelepin, Vershinina, & Novikova, 2015), vernier acuity (Keri, Kelemen, Benedek, & Janka, 2004), forward and backward masking (Green, Lee, Wynn, & Mathis, 2011), flanker effects (Keri, Kelemen, Benedek, & Janka, 2005), surround suppression (Dakin, Carlin, & Hemsley, 2005), size estimation (Asarnow & Mann, 1978), distance estimation (Weckowicz, Sommer, & Hall, 1958), perceptual organization (Silverstein & Keane, 2011), coherent motion perception (Chen, 2011), face perception (Turetsky et al., 2007), size constancy (Silverstein et al., 2013), and effects of prior knowledge on interpretation of visual input (Keane, Silverstein, Wang, & Papathomas, 2013). In addition, 25%–30% of individuals with schizophrenia report visual hallucinations (Waters et al., 2014), and the rate of patients reporting visual distortions (in the domains of brightness, motion, form, and color) has been estimated to be from 30% to over 60% (Bunney et al., 1999; Cutting & Dunne, 1986; Phillipson & Harris, 1985). These findings raise the question of whether each of these impairments reflects a unique mechanism or whether in fact there are a relatively small number of processing disturbances whose expressions, either alone or in combination, can manifest in multiple ways, depending on task conditions and demands. The latter suggestion is consistent with the concept of canonical cortical computations, or core processing strategies, that are common to cortical regions and that contribute to, or primarily drive, multiple cognitive and perceptual phenomena (Carandini & Heeger, 2011; Phillips, Clark, & Silverstein, 2015; Phillips & Silverstein, 2003). For example, in the area of visual disturbances in schizophrenia, it has been proposed that changes in gain control and integrative mechanisms can account for many of the laboratory findings across multiple studies and paradigms (Butler, Silverstein, & Dakin, 2008). More recently, it was shown that disturbances in contextual modulation, including combinations of its subprocesses of amplification, suppression, and synchronization, can account for most of the laboratory and phenomenological visual processing disturbances in schizophrenia that are noted earlier (Silverstein, 2016). However, no matter how well new findings from cognitive neuroscience can be used to explain older data, such arguments are essentially theoretical and are not as strong as new findings demonstrating that manipulations of proposed mechanisms lead to predicted effects. Therefore, in this article, we apply computational modeling to determine whether changes to a small number of mechanisms can simulate two aspects of visual perceptual functioning that have not been previously addressed in the literature: (a) opposite findings on CS in unmedicated first-episode versus chronic schizophrenia patients and (b) findings of both reduced CS and broadened orientation (OR) tuning in chronic schizophrenia. Each of these issues is briefly reviewed in the following paragraphs.

Studies of CS in chronic schizophrenia have generally reported reduced CS at low SFs and marginally reduced CS at higher SFs (O’Donnell et al., 2006; Slaghuis, 1998). However, these studies have varied in terms of stimuli, presentation parameters, and duration of illness, all of which are factors that affect the strength of between-group differences. Some studies have found impairments in chronic schizophrenia that are limited primarily to low SFs, with normal processing of other SFs (Butler et al., 2007). Others have argued that, across all studies, the data are most consistent with reduced sensitivity to all contrasts and SFs (Skottun & Skoyles, 2007), suggesting a more general problem with a weakening of response gain mechanisms as the illness progresses (Butler et al., 2008; Herzog & Brand, 2015; Shelley, Silipo, & Javitt, 1999; Silverstein, 2016; Skottun & Skoyles, 2013). For the purposes of this article, we adopt the intermediate position that the CS deficit in chronic schizophrenia is largest for low SFs but that processing of medium and high SFs is impaired to some degree as well. However, we believe that impairment at higher SFs is due primarily to limitations in visual acuity and not to problems in CS per se. Evidence for this is that even in healthy observers, CS declines at higher SFs (i.e., the CS function begins to resemble an inverted U-shaped curve as SFs greater than six cycles/degree are tested, and neural activity declines in parallel; Goodyear, Nicolle, Humphrey, & Menon, 2000). Because individuals with schizophrenia are known to have poorer visual acuity than the general population (Silverstein & Rosen, 2015; Viertio et al., 2007), they would be expected to show poorer CS than controls at higher SFs. Although we do believe that there are problems in response gain in schizophrenia, and that they may contribute to problems processing high-SF stimuli, we do not believe this is the primary contributor to impaired high-SF processing. This is because if the issue were primarily response gain, then CS might be expected to be *differentially worse* at high SFs relative to low SFs, owing to the stronger signaling and wider range of possible values with higher SFs, but this has never been reported (and nearly all studies have reported the opposite). In short, the goal of our chronic schizophrenia modeling efforts was to reproduce a large impairment, relative to controls, at a low SF, and a small impairment at a higher SF. In contrast, we attempted to model an *increase* in CS for low SFs and normal CS at medium SFs in unmedicated first-episode schizophrenia (FES), as has now been demonstrated psychophysically in several studies (Cadenhead, Dobkins, McGovern, & Shafer, 2013; Kelemen, Kiss, Benedek, & Keri, 2013; Kiss, Fabian, Benedek, & Keri, 2010; Shoshina et al., 2015).

The second pattern of findings we attempted to simulate was the combination of both reduced CS (as noted earlier) and broadened OR tuning (Rokem et al., 2011; Schallmo, Sponheim, & Olman, 2013) in chronic schizophrenia. Although both phenomena have been demonstrated in chronic patients, no study has tested both processes in the same patients. Therefore our goal was to see whether the same mechanisms involved in reducing CS would also broaden OR tuning.

## METHODS

### Modeling Environment

All models were run using the Topographica simulator (Bednar, 2009, 2012; Bednar, Kelkar, & Miikkulainen, 2004), which is freely available at https://github.com/ioam/topographica, with documentation at http://www.topographica.org/. Topographica was developed for modeling the development of cortical maps and has typically been used for computational modeling of aspects of low- and mid-level vision (e.g., orientation preference maps, orientation tuning, contrast sensitivity, aftereffects, illusions, perceptual organization).

### Baseline Model Characteristics

**Figure 1.**
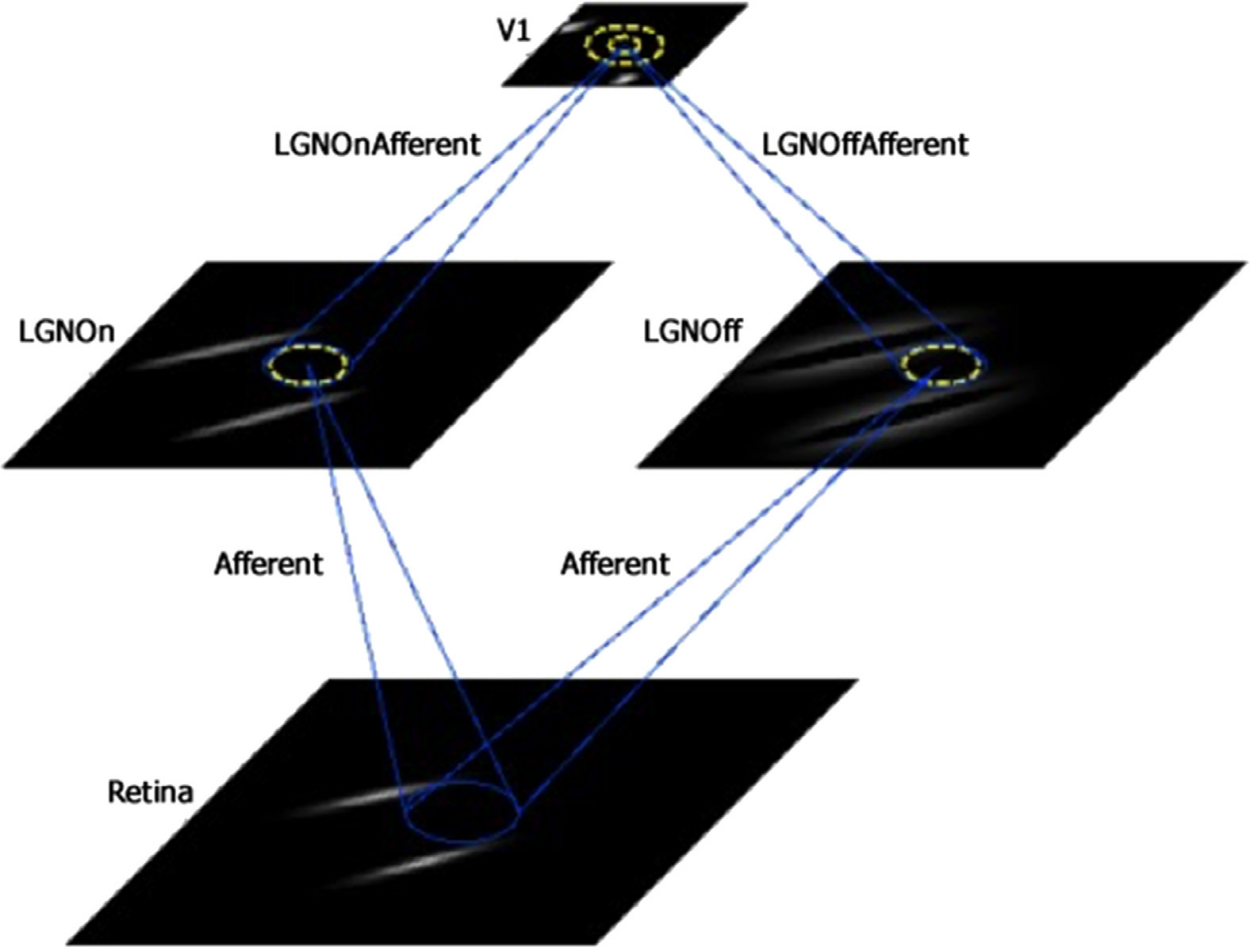
**Depiction of the sheets and connections in the gain control, adaptation, laterally connected (GCAL) class of models used in this study.** Sheets include retina, lateral geniculate nucleus (LGN) On and LGN Off, and V1. Projections include afferent input to each of the LGN sheets and afferent input to V1 from each of the LGN sheets as well as lateral excitatory feedback within V1 (inner yellow circle) and a wider range of lateral inhibitory feedback within V1 (outer yellow circle). Examples of the training stimuli used in each model (i.e., pairs of oriented Gaussians) can be seen in the retinal sheet, with corresponding transformations in the LGN and V1 sheets.

For this project, we used as our baseline the gain control, adaptation, laterally connected (GCAL) model (Stevens, Law, Antolik, & Bednar, 2013; further described in Bednar, 2012, 2014a, 2014b; Bednar & Wilson, 2016). GCAL incorporates several features that are biologically realistic and reasonably noncontroversial but that, for the most part, were not included in older self-organizing map (von der Malsburg, 1973) models (Miikkulainen, Bednar, Choe, & Sirosh, 2005). These include (a) gain control at the LGN level, (b) homeostatic adaptation of V1 responses based on a weighted sum of all inputs and limited by a logistic (sigmoid) nonlinearity, and (c) weights on excitatory and inhibitory lateral connections within V1 and on afferent connections to V1. These weights begin as isotropic (radially uniform) but subsequently modify in a self-organizing fashion upon repeated presentations of visual input and other forms of neural activity according to Hebbian (unsupervised activity-dependent) learning, with divisive normalization. Other characteristics of the GCAL model, which are shared with some prior models, include the assumption of single-compartment firing-rate neurons at the retinal ganglion cell, LGN, and V1 levels; hard-wired subcortical pathways to V1, including the main types of LGN neurons (e.g., On center–Off surround; Off center–On surround); roughly retinotopic projections from the retinal to the LGN sheets to the V1 sheet; and separate parameters for excitatory and inhibitory activity (see Figure 1). GCAL has successfully modeled a wide range of phenomena expressed in V1 (e.g., development of contrast-invariant orientation tuning and direction selectivity; development of ocular dominance, aftereffects, and surround suppression effects). Such modeling results show that these effects can be explained by a small number of canonical mechanisms (Bednar, 2014a, 2014b). These demonstrations also indicate that through visual experience, the statistical regularities of the environment are learned and encoded via the competitive processes inherent to Hebbian learning and that these processes are constrained by gain control and homeostatic mechanisms to prevent runaway neural excitation in frequently activated circuits.

The basic GCAL model has four levels or sheets, each of which is implemented as a two-dimensional array of firing-rate neurons: a retina (24 × 24 density), LGN On and Off channel sheets (24 × 24 density), and a V1 sheet (48 × 48 density; see Figure 1). Here *density* represents the number of simulated units (neurons) per unit area of the indicated sheet, corresponding to a square portion of the simulated visual field. The retinal and LGN sheets thus have one-fourth the number of units per visual area as does the V1 sheet. Note that in this model, the retinal sheet is equivalent to the photoreceptor layer of the retina only. The model LGN sheet activity is an abstraction that represents all levels between the photoreceptors and the superficial layers of V1 that combine and transform the photoreceptor signals, including the retinal bipolar and ganglion cell layers and the LGN itself. As illustrated in Figure 1, these four sheets are interconnected via sets of projections, or *connection fields*, whose synaptic weights on the next sheet level are modifiable by Hebbian learning and by changes in learn ing rates.

In a typical model, activity is simulated in a series of time steps, with step size *δt* = 0.05, which corresponds to approximately 12.5 milliseconds in real time. At time *t* = 0.0, the image reaches the retina; at time *t*+0.05, the LGN On and Off sheets calculate their responses; at time *t*+0.10, the output of the LGN On and Off sheets reaches the V1 sheet; and from times *t*+0.15 to *t*+0.95, the activity within the V1 sheet propagates and settles through lateral inhibi tory and excitatory connections. At *t* = 1.0, the next stimulus is presented to the retina, and this continues for the number of iterations chosen by the user. Details of model functioning are reported in supplemental Appendix A (Silverstein, Demmin, & Bednar, 2017).

### Model Parameter Manipulations

For the purposes of this project, we chose to manipulate eight parameters that reflect known or hypothesized changes in the visual system in schizophrenia (see Table 1). These manipulations can be characterized as falling into three types of changes in brain function: (a) changes in input strength, (b) changes in lateral excitation and inhibition, and (c) changes in plasticity. Changes in input strength included manipulations such as decreased retinal input to LGN, decreased LGN contrast gain control (which would lead to changes in input level to V1), and decreased LGN input to V1. Changes in excitation and inhibition included increased lateral excitation within V1, reduced lateral inhibition within V1, and a reduced homeostatic adaptation rate within V1 (allowing for sustained increased firing within V1). Changes in plasticity included an increased afferent learning rate at LGN-V1 connections and an increased excit atory learning rate within V1. Each of these is briefly described in the following sections, along with a rationale for the manipulation.

**Table 1.** Summary of gain control, adaptation, laterally connected (GCAL) parameters manipulated in the schizophrenia models

**Parameter manipulated**	**Effect**
Input strength	Decreased retinal input to LGN
	Decreased LGN contrast gain control
	Decreased LGN input to V1
Excitation and inhibition	Increased lateral excitation within V1
	Reduced lateral inhibition within V1
	Reduced homeostatic adaptation rate within V1
Plasticity	Increased afferent learning rate at LGN-to-V1 connections
	Increased excitatory learning rate within V1

*Note*. LGN = lateral geniculate nucleus.

### Changes in input strength

#### Decreased afferent input to LGN (On and Off)

Several lines of evidence indicate that afferent input to the LGN is reduced in schizophrenia. First, schizophrenia is associated with reduced retinal photoreceptor and bipolar cell signaling, as indicated by smaller flash electroretinogram (ERG) waveform amplitudes (Balogh, Benedek, & Keri, 2008; Hebert et al., 2010; Hebert et al., 2015; Lavoie, Maziade, & Hebert, 2014; Silverstein & Rosen, 2015), and this change is more pronounced in patients at the outset of treatment for an acute psychotic episode than it is after several weeks of treatment (Balogh et al., 2008). Second, reduced retinal signaling could be expected to reduce the strength of LGN output and therefore the strength of activity reaching V1, and indeed, reduced amplitudes of the visual evoked potential (VEP) have been repeatedly observed in schizophrenia, with evidence that the problem is more pronounced in unmedicated patients (Shagass & Roemer, 1991; Shagass, Straumanis, & Roemer, 1982; Straumanis, Shagass, & Roemer, 1982). Third, multiple lines of evidence indicate that schizo phrenia is characterized by dopamine (DA) dysfunction (Howes & Kapur, 2009; Kegeles et al., 2010; Slifstein et al., 2015), including elevated presynaptic striatal DA functioning. There is reason to believe that DA dysregulation may also be found in the retina in schizophrenia, because excess retinal DA would have the effect of suppressing rod photoreceptor activity (Brandies & Yehuda, 2008; Witkovsky, 2004), which is consistent with ERG data in newly treated patients. Excess retinal DA would also have the effect of uncoupling horizontal cell activity, thereby further reducing the strength of retinal output (Silverstein & Rosen, 2015). On the basis of all of these considerations, and especially those of more severe ERG waveform amplitude attenuation in untreated patients, the partial normalization of ERG data from treatment with DA antagonists, and suggestions of more severely attenuated VEPs and excessive retinal DA in first-episode patients, we developed models that included reduced afferent input to V1 (i.e., reduced retinal and also LGN efferents; see later). Furthermore, we hypothesized that these changes would generate data resembling those of unmedicated FES (but not chronic [medicated]) patients, namely, increased CS for low-SF stimuli and normal CS for medium-SF stimuli.

#### Decreased LGN (On and Off channels) contrast gain control

The strength of LGN lateral inhibitory projections implementing contrast gain control in the LGN was decreased by varying degrees, across multiple models, as the sole manipulation, or in combination with the perturbations that provided the best fit to the chronic schizophrenia and FES data. This set of models was run to determine if the gain control impairments hypothesized to exist in schizophrenia (Butler et al., 2008; Butler et al., 2005; Silverstein, 2016) are likely to occur as early as the LGN.

#### Decreased LGN (On and Off channels) input to V1

Afferent input to V1 was reduced by varying degrees based on generally consistent findings of reduced VEP amplitude in schizophrenia, as noted earlier (Butler et al., 2005; Schechter et al., 2005). Because we assumed that reduced afferent input to LGN channels (see Decreased Afferent Input to LGN [On and Off]) would be associated with further reduction of LGN output, even with the existence of gain control mechanisms within the LGN channels, we reduced afferent strength to V1 more in the FES than in the chronic models.

### Changes in excitation and inhibition

#### Increased lateral excitation within V1

Two sources of evidence suggest elevated local excit atory activity within V1. One is the similarity between hyperglutamatergic effects of ketamine administration in healthy volunteers and brain function in schizophrenia (Anticevic et al., 2013; Anticevic et al., 2015; Corlett, Honey, Krystal, & Fletcher, 2011). The second is evidence for elevated baseline gamma-band power and synchrony in people with schizophrenia (Rivolta et al., 2014; Silverstein et al., 2012; Sun et al., 2013), suggesting abnormal network formation (see also Increased Excitatory Learning Rate Within V1).

#### Reduced lateral inhibition within V1

The strength of inhibition within V1 was reduced based on findings of reduced GABA concentration in the visual cortex of people with schizophrenia (Kelemen et al., 2013; Thakkar et al., 2016; Yoon et al., 2010) as well as on findings of broadened OR tuning in cats after administration of the selective GABA_A_ antagonist gabazine (Katzner, Busse, & Carandini, 2011).

#### Reduced homeostatic adaptation rate within V1

The homeostatic adaptation rate refers to the speed with which systems that balance excitation and inhibition operate to bring highly (or weakly) active neurons back to their target firing rate (Davis & Bezprozvanny, 2001; Turrigiano, 2011; Xue, Atallah, & Scanziani, 2014). Reducing this value allows for longer than normal elevations in firing rate prior to return to the target rate. These manipulations were considered exploratory because while such a change would fit with prior clinical reports of perceptual disturbances in schizophrenia that include increased stimulus intensity (e.g., in brightness or color), especially in FES patients, no study, to our knowledge, has demonstrated a slower rate of return to homeostasis in schizophrenia.

### Changes in plasticity

#### Increased afferent learning rate at LGN-to-V1 connections

This manipulation to the Hebbian learning rate was based on findings of reduced reliability in neuronal coactivation patterns in schizophrenia (Hamm, Peterka, Gogos, & Yuste, 2017) and tighter coupling between thalamic and cortical sensory processing regions in schizophrenia compared to healthy controls (Anticevic et al., 2014). Because it has not actually been shown that there is increased Hebbian learning in V1 in schizophrenia, however, this set of manipulations was exploratory and more speculative than the others described earlier.

#### Increased excitatory learning rate within V1

The speed with which weights at excitatory connections within V1 were updated was increased to varying degrees, to explore one potential effect of increased excitatory activity within V1. This was based on evidence of (a) hyperactivation of the locus coeruleus (LC) in schizophrenia (Alsene & Bakshi, 2011; Yamamoto & Hornykiewicz, 2004) and modeling data suggesting that elevated LC activation increases both gain and the rate of Hebbian learning (Verguts & Notebaert, 2009); (b) modeling data demonstrating that as cortical activity levels increase, membrane time constants are lowered, leading to increases in synchronized firing between cells and to aberrant local network formation (Chawla, Lumer, & Friston, 1999); and (c) evidence from animal studies that excess neuronal firing can lead to the formation of aberrant cell assemblies (Olypher, Klement, & Fenton, 2006; Sun et al., 2013), a process that has been hypothesized to occur in schizophrenia (Hoffman & McGlashan, 1993).

### Model Training and Modeling Strategy

The basic modeling strategy was first to develop a normally functioning visual cortex by presenting 10,000 pairs of stimuli (see Figure 1) to a model designed to mimic normal development of topographic maps in V1 based on lateral connectivity, Hebbian learning, gain control, and homeostatic mechanisms. Then, after 10,000 iterations of stimulus presentation, we changed one or more parameters to simulate a known or hypothesized change in schizophrenia. We then ran the modified model for 10,000 additional iterations, after which we examined the effects of the change(s) made at iteration 10,001 on the dependent variables (see next paragraph), relative to an unmodified (control) model where the visual system was trained with 20,000 iterations without any perturbations. These values were chosen to ensure consistency with past studies using the GCAL model, but also because (a) 20,000 iterations can be viewed as a point of complete development, at which receptive fields are orientation selective and a very stable topographic organization for orientation is observed (Stevens et al., 2013); (b) even at 10,000 iterations, significant stability is observed (Bednar, 2014a, 2014b); and (c) we wanted to simulate schizophrenia-related changes as occurring at a point corresponding to late adolescence or early adulthood, so we chose values of 10,000 for the onset of changes and 20,000 as the point at which the effects of the changes were described. Note that while this allowed for significant time for the effects to evolve, we also demonstrated (see Single Best Fitting Model) that the best fitting model for FES could be replicated in as few as 1,000 additional iterations after the appropriate changes were made at 10,000 *or* at 20,000. Moreover, data simulating findings from chronic schizophrenia were observed whether the model was run to 10,000 or 20,000 before making changes and running it for another 10,000 iterations. These findings confirm the acute nature of the FES effects and the robustness of the chronic schizophrenia effects.

**Figure 2.**
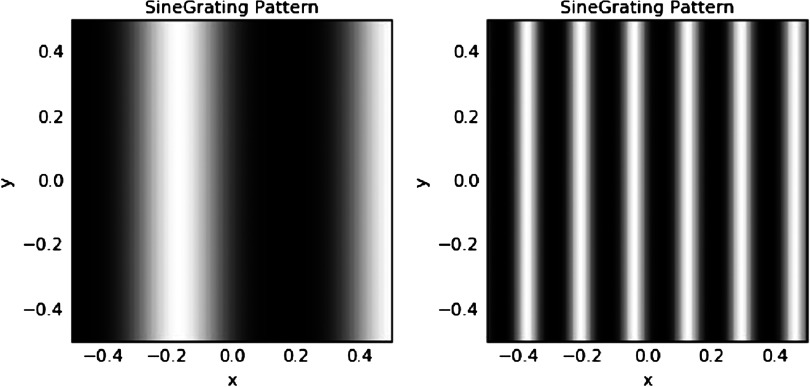
**Stimuli used for posttraining testing.** Left: low-spatial-frequency (SF) stimulus (frequency = 1.5 cycles per image). Right: medium-SF stimulus (frequency = 6 cycles per image).

After each model was *developed* with 20,000 iterations, as described previously, its functioning was *tested* by presenting it with a different set of stimuli. The postdevelopment stimuli consisted of sinusoidally modulated vertical gratings^1^ at low and medium SFs, each of which was presented at five levels of contrast (5%, 10%, 20%, 40%, and 80%; see Figure 2), defined as the percentage of the possible input range, from 0.0 to 1.0 (Stevens et al., 2013). The primary dependent variables for analyses regarding CS were the mean level of V1 activation for each of the posttraining test stimuli in each of the contrast * SF conditions.^2^ Regarding orientation tuning, broadening was defined by the presence of one or more of the following: flattening at the peak of the orientation tuning curves, bimodally peaked distributions, excess kurtosis values indicating greater than expected activity at the tails, and/or shifts in the peak of the orientation tuning histogram away from the expected value of *π*/2 rad (90°, or vertical). For the simulations reported in the following pages, data are reported primarily in terms of mean activation levels as well as graphically to show effects such as shifts or shape changes in orientation tuning curves. Graphics include levels of retinal, LGN, and V1 activation; orientation preference plots; combined orientation preference and activation maps; and orientation preference histograms.

An assumption common to all models presented is that higher V1 activation values to the sinusoidal gratings correspond to increased sensitivity of an observer in a psychophysical task measure of CS. This assumption is supported by several lines of evidence. One is that, using single-unit microelectrode recording in cat V1, the psychophysical CS function (CSF) was highly correlated with the neuronal CSF (Meng et al., 2013). Another is that the fMRI BOLD response in humans covaries with contrast enhancement (Boynton, Demb, Glover, & Heeger, 1999) and SF frequency (Goodyear et al., 2000), with the relationship being especially tightly coupled for low-SF stimuli (Olman, Ronen, Ugurbil, & Kim, 2003). On the basis of this evidence, we assumed that reduced activation would be associated with decreased CS.

### Hypotheses and Planned Simulations

One of the hypotheses we explored was that decreased V1 inhibition, as shown previously in chronic schizophrenia (Dakin, Carlin, & Hemsley, 2005; Schallmo, Sponheim, & Olman, 2015; Silverstein, 2016), and as a presumed consequence of reduced V1 GABA concentration (Kelemen et al., 2013; Thakkar et al., 2016; Yoon et al., 2010), would contribute to both the reduced CS and the broadened OR tuning that have been observed in chronic schizophrenia. Other parameter changes (e.g., in the V1 excitatory learning rate) were added to reduced inhibition in an exploratory fashion to determine if these led to a better fit to previously reported data than reduced V1 inhibition alone. Our specific hypotheses regarding *chronic schizophrenia* were that (a) decreased CS, at the level of a 20%–35% reduction in CS at low SFs (O’Donnell et al., 2006; Shoshina et al., 2015), would emerge as a result of decreased V1 inhibition; (b) broadened OR tuning, at the level of ∼20%, would also emerge as a result of reduced V1 inhibition, based on findings of both reduced V1 GABA concentration and of this level of broadened OR tuning in chronic schizophrenia patients (Rokem et al., 2011), as well on known effects of GABA antagonists on OR tuning (Katzner et al., 2011); and (c) further broadening in OR tuning would emerge with increases in the Hebbian learning rate for weights of afferent input to V1 and/or excitatory influences within V1.

As noted, we predicted that reduced retinal signaling would be related to the emergence of elevated CS in FES patients for low-SF stimuli. Our goal was to develop a model in which reductions in retinal signaling would be associated with an ∼25% increase in CS for low-SF stimuli only. While studies of CS in unmedicated FES have varied in their experimental methods and findings, we chose to fit the pattern demonstrated by Shoshina et al. (2015) because (a) that study used stimuli similar to those used in our models; (b) it had a larger number of untreated first-episode patients (*n* = 11) than the study of Cadenhead et al. (2013; *n* = 5), which examined medication effects in post hoc analyses only; and (c) while it reported a smaller increase in CS compared to Kelemen et al. (2013; *n* = 28), who reported an ∼33% increase, the stimuli and presentation methods used in the latter study differed significantly from those used in our models (in terms of SFs and stimulus backgrounds), and we also wished to be conservative and assume a smaller between-group effect, given the small number of (mostly small) studies of this issue.

We also explored the extent to which adding cortical changes to the FES model, such as reduced inhibition or increases in the afferent learning rate, brought the simulated findings closer to previously published findings. As noted, there are no published reports on OR tuning in FES, and so we also explored whether the factors that would lead to broadened tuning in chronic patients could be added to models producing the FES pattern of CS findings without significantly altering those CS findings.

## RESULTS

Results are organized as follows. First, characteristics of the unmodified GCAL model, after 20,000 iterations, are presented in the text, in the first panel showing data in each of the figures, and by the thick line in each line graph. Second, effects of changes in the model parameters discussed earlier are briefly summarized. Third, the best fitting models for the chronic and FES groups are described in detail, whereas parameter manipulations that did not lead to good model fits are described in supplemental Appendix B (Silverstein et al., 2017). Data from the schizophrenia models are presented in each of the figures in panels to the right of the data from the unmodified GCAL model or by thin colored lines in each line graph. A summary of all of the models tested and the consistency of their output with published findings is presented in Tables 1 and 2.

**Table 2.** General classes of models run in the schizophrenia simulations and summary of the fit of models to chronic schizophrenia and/or first-episode schizophrenia data

**Model Classes**	**Result** ^**a**^
Increased V1 lateral excitation (range = 10% to 50%)	None
Reduced V1 lateral inhibition (range = 10% to 50%)	None
Increased V1 lateral excitation (20%) +	
Increased afferent learning rate at V1 (up to 3×)	None
or - Reduced V1 homeostatic adaptation rate (up to 90% *↓*)	None
or - Increased V1 excitatory learning rate (0.0 to 0.2)	None
Reduced V1 lateral inhibition (10%) +	
Increased afferent learning rate at V1 (up to 3×)	Chronic^b^ (best fit)
or - Reduced V1 homeostatic adaptation rate (up to 90% *↓*)	Chronic (CS but not orientation tuning)
or - Increased V1 excitatory learning rate (0.0 to 0.2)	Chronic (second best fit)
Reduced retinal input to LGN and reduced LGN input to V1 (range = 10% to 50%) +	FES^c^
each of the manipulations described above	No significant improvement^d^ over reduced retinal and LGN afferents alone
Reduced LGN contrast gain control (range = 25% to 75%)	None

*Note.* FES = first-episode schizophrenia. LGN = lateral geniculate nucleus.

^a^(i.e., fit for Chronic, FES, or none). ^b^At 10% reduced V1 lateral inhibition and 3× increased afferent learning rate at LGN synapses onto V1. ^c^With 15% reduction in both variables. ^d^Increasing the afferent learning rate on LGN synapses to V1 led to broadened orientation tuning (an issue that has not yet been studied in FES) while leaving increased CS for the LSF stimulus unaffected.

### Baseline Model Characteristics

**Figure 3.**
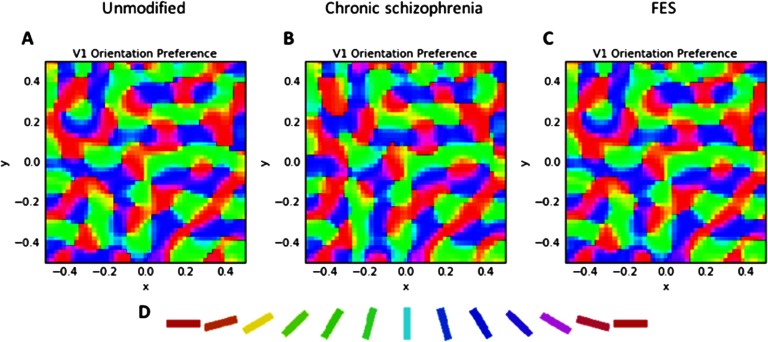
**V1 orientation preference maps.** A) The unmodified GCAL model after 20,000 iterations. B) The best fitting model for chronic schizophrenia data, involving 10% reduced V1 lateral inhibition and a tripled V1 afferent learning rate at iteration 10,001, with the model then run for another 10,000 iterations. C) The best fitting model for first-episode schizophrenia (FES) data, involving 15% reduced strength of retinal and LGN efferent activity at iteration 10,001, with the model then run for another 10,000 iterations. The qualitative similarity between the maps AC indicates that, despite perturbations to schizophrenia-relevant parameters that led to increases in contrast sensitivity (CS) (in C) and reduced CS and broadened orientation tuning (in B), realistic topography of orientation selectivity in V1 was maintained. D) Orientation key. Each color in the maps corresponds to maximum selectivity for the orientation denoted by the corresponding color in the key.

After 20,000 iterations, the unmodified GCAL simulation produced maps and statistics that replicated those observed in earlier demonstrations (Bednar, 2014a, 2014b; Stevens et al., 2013). Figure 3A indicates that a smooth topographic map of orientation preferences emerged from the simulation, which, like past simulations with GCAL, is similar to maps from normally developing animals from several mammalian species. Similar maps for orientation are expected in humans but have not been measured, because doing so would require invasive imaging techniques.

**Figure 4.**
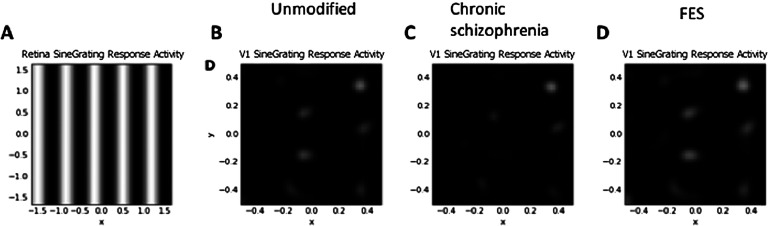
**V1 retinotopic activation maps in response to the low-SF stimulus at 80% contrast.** A) The stimulus, here compressed to show over a larger spatial extent, and so SF appears higher than in the actual stimulus, shown in Figure 2. B) Unmodified GCAL model after 20,000 iterations (mean activation = 0.029; max. = 0.512). C) The best fitting model for chronic schizophrenia data (mean = 0.007; max. = 0.386). D) The best fitting model for FES data (mean = 0.041, max. = 0.540). Greater activation is indicated by increased brightness. Reduced activation to the low-SF stimulus can be seen in C, the chronic schizophrenia model. Increased activation to the same stimulus can be seen in D, in the FES model. These changes, relative to the unmodified model (B), and differences from each other are consistent with published data on CS in these groups.

**Figure 5.**
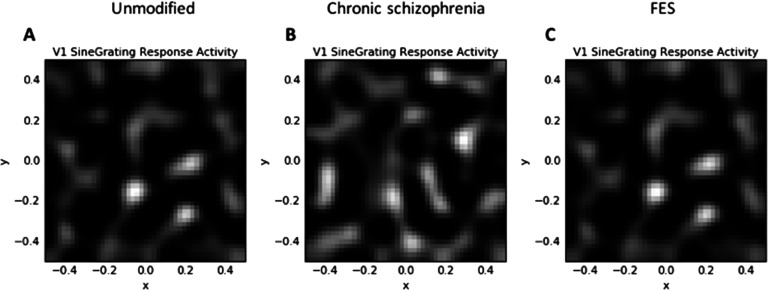
**V1 retinotopic activation maps showing response to the medium-SF stimulus at 80% contrast.** A) The unmodified GCAL model after 20,000 iterations (mean activation 0.137; max. = 0.587). B) The best fitting model for chronic schizophrenia data (mean activation = 0.140, max. = 0.550). C) The best fitting model for FES data (mean activation = 0.131; max. = 0.564). Note that, as predicted, at this higher SF, activation is higher than shown in Figure 4. Also note that a greater spread of activation can be seen in the chronic schizophrenia model compared to the other models (i.e., there is activation of orientation-selective simple cells that are not activated in the other models). This greater activation of orientation-selective cells that are normally relatively silent may be the basis of broadened orientation tuning in this group.

**Figure 6.**
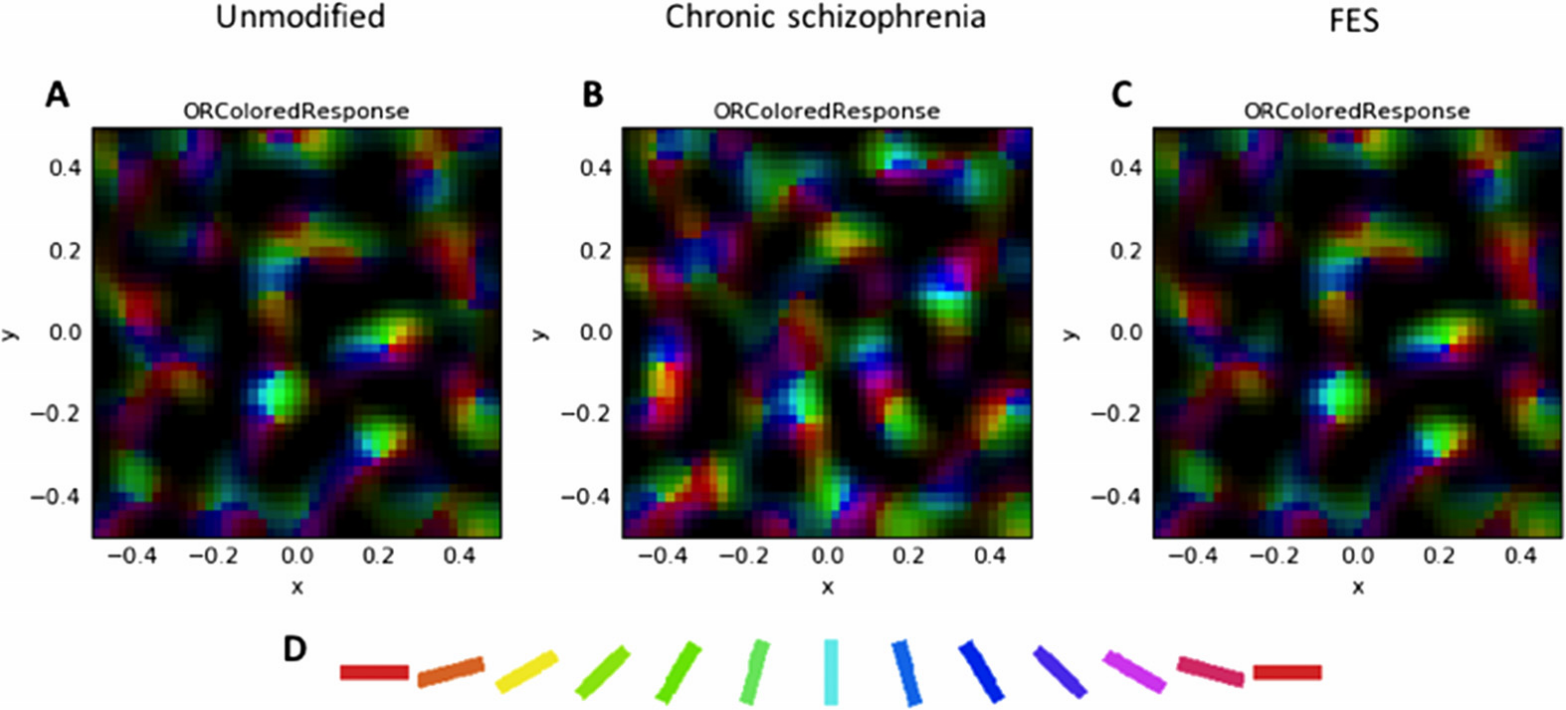
**Combined orientation preference and activation maps showing response to the medium-SF stimulus at 80% contrast.** A) The unmodified GCAL model after 20,000 iterations. B) The best fitting model for chronic schizophrenia data. C) The best fitting model for FES data. It can be seen here that the broader orientation tuning in the chronic schizophrenia model (B; see Figure 5) arises owing to greater activation of cells not selective for a vertical orientation (see D for orientation key).

**Figure 7.**
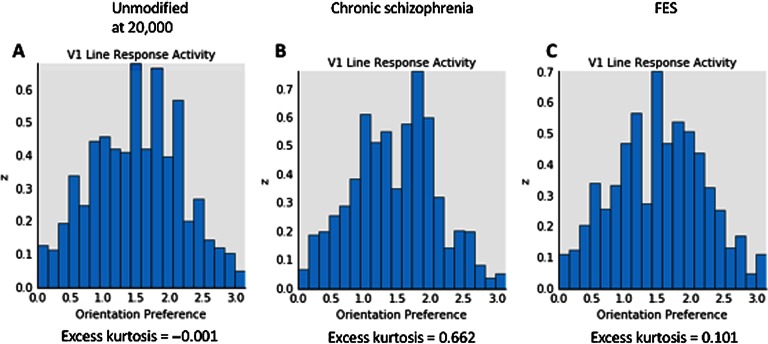
**Orientation tuning histograms (V1 orientation map weighted by the strength of activation of each unit) showing responses (*y* axis, in arbitrary units) to the medium-SF stimulus at 80% contrast.** A) The unmodified GCAL model after 20,000 iterations. B) The best fitting model for chronic schizophrenia data. C) The best fitting model for FES data. The *x* axis depicts orientation in radians. The unmodified model (A) shows a typical Gaussian curve with the peak orientation at vertical (90° or *π*/2 or ∼1.57 rad). In the chronic schizophrenia model (B), there is suppression of cells signaling this orientation and a near to bimodal distribution with separated peaks at both lesser and greater orientations, which may be the basis for broadened orientation tuning in this population. The high excess kurtosis value for this panel indicates that the chronic model is also associated with the greatest activation in neurons signaling orientations far from the peak. In the FES model (C), the peak response is to a vertical stimulus, and the shape of the distribution is essentially Gaussian. Note that in models B and C, there is slightly greater peak activation for the peak response compared to model A, as indicated by the larger range on the *y* axis.

**Figure 8.**
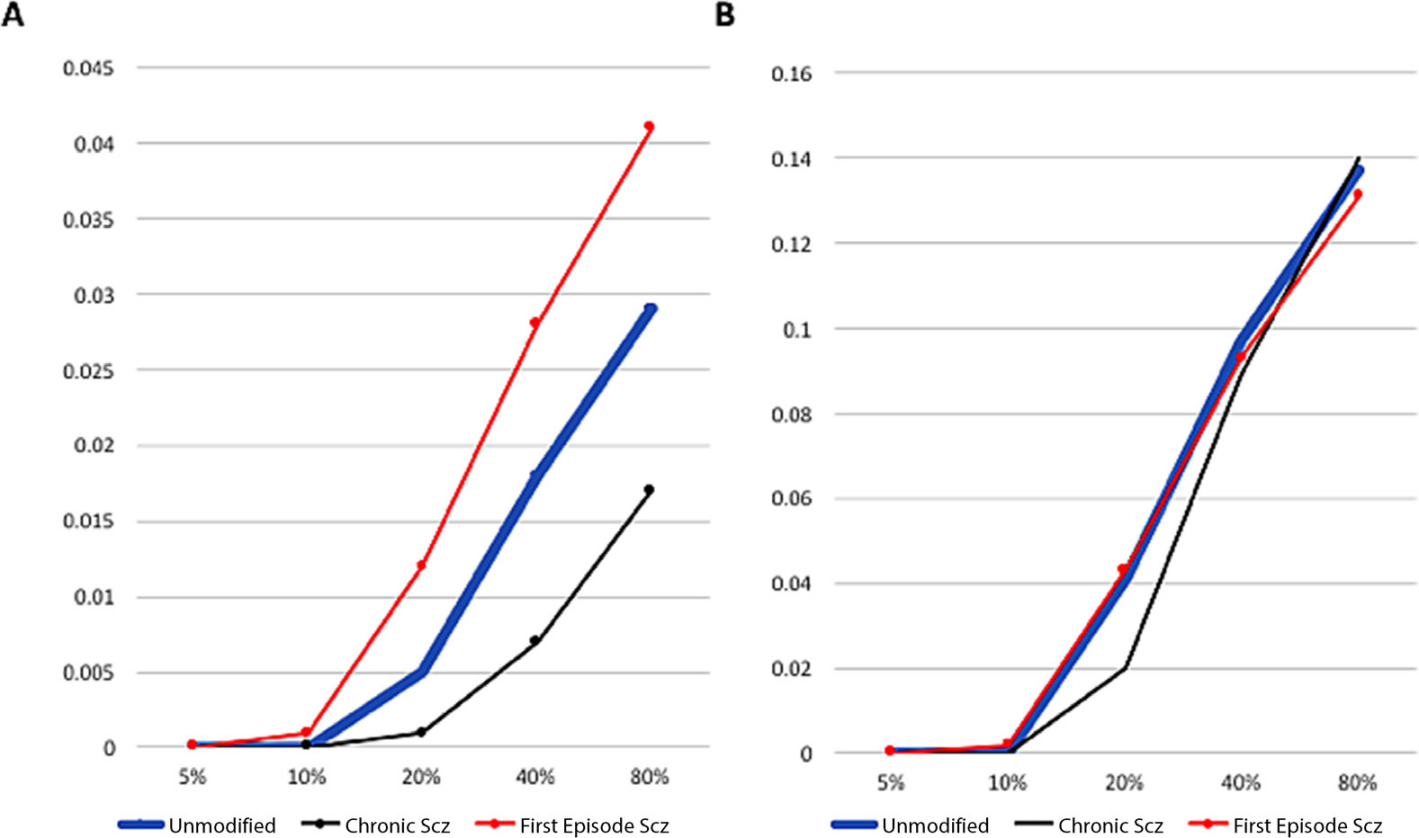
**Activation (*y* axis, in arbitrary units) values across contrast levels (*x* axis) for the unmodified GCAL model, the best fitting chronic schizophrenia model, and the best fitting FES model.** A) The low-SF stimulus. B) The medium-SF stimulus. In both panels, the unmodified model is represented by the thick blue line. The pattern displayed here is one of (a) increased activation in the FES model, and reduced activation in the chronic schizophrenia model, in response to the low-SF stimulus and (b) normal activation in the FES model, and slightly reduced activation at medium contrast levels in the chronic schizophrenia model, in response to the medium-SF stimulus, consistent with published observations for these patient groups. Note that the activation range is higher for the medium-SF stimulus, as indicated by the *y* axis in B.

Figure 4B depicts V1 activation after presentation of the low-SF stimulus at 80% contrast. This also depicts the normal patchy pattern of orientation selectivity in V1. Figure 5A depicts activation levels in V1 for the medium-SF stimulus at 80% contrast. This figure, relative to the V1 sheets in Figure 4, demonstrates the normal greater spread of activation with higher SF stimuli. Figure 6A demonstrates that most of the regions of V1 activation involve cells tuned to vertical (90°) stimuli, again indicating normal map development. Figure 7A displays the distribution of activation levels for cells of all possible orientations, immediately after measurement of the orientation map, and demonstrates again that the strongest signal was for vertically oriented stimuli. The low excess kurtosis value of − 0.001 indicates that the orientation tuning curve closely follows a normal distribution and that there was not greater than expected activity in orientation-selective cells tuned for highly nonvertical orientations. Finally, Figures 8A and 8B demonstrate that activation increased in the typical nonlinear fashion with increases in contrast (plotted on the *x* axis) and that there was greater activation for higher than there was for lower SF stimuli (as can be seen by the differences in *y* axis range from Figure 8A to Figure 8B).

### Schizophrenia Model Characteristics

**Figure 9.**
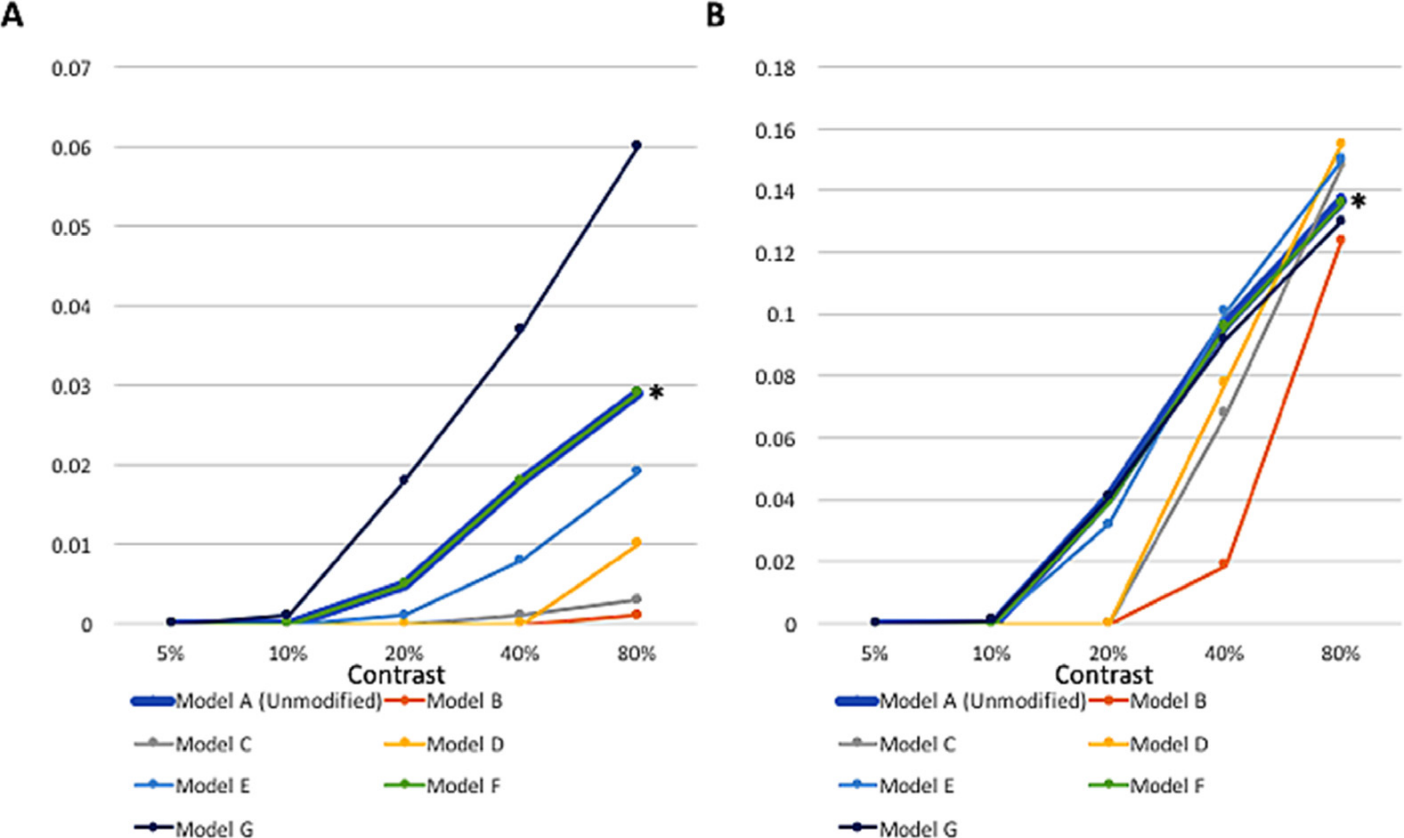
**Activation values (*y* axis, in arbitrary units) across contrast levels for the unmodified GCAL model, after 20,000 iterations, and several alternative schizophrenia models discussed in the text.** Left: activity in response to the low-SF stimulus. Right: activity in response to the medium-SF stimulus. Model A (unmodified), represented by the thick blue line, is the basic GCAL model. All other models represent cases with perturbations at the 10,001st iteration, as follows: Model B, 25% increase in V1 lateral excitation; Model C, 25% reduction in V1 lateral inhibition; Model D, 20% increase in V1 lateral excitation and an increase in the V1 excitatory learning rate to 0.01; Model E, 10% reduction in V1 lateral inhibition and an increase in the V1 excitatory learning rate to 0.01; Model F (barely visible owing to almost complete overlap with Model A, but indicated by an asterisk), an increase in the V1 afferent learning rate to 0.03 without any change to lateral inhibition; Model G, 15% reduction in both retinal and LGN efferents in combination with an increase in the V1 afferent learning rate to 0.03. Note that although Models G in the left panel and F in the right panel are reasonable fits to previously published FES CS data, and although Models E in the left panel and C and D in the right panel are reasonable fits to previously published chronic schizophrenia data, none of these models are a good fit across *both* the left and right panels. Results for these same models are also depicted in Figure 10.

**Figure 10.**
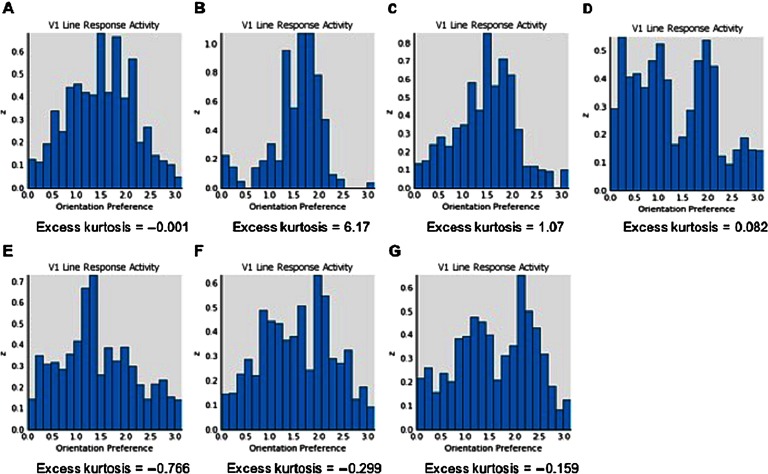
**Orientation tuning histograms showing response to the medium-SF stimulus at 80% contrast for Models A-G (as described in Figure 9).** Although Models DG would be expected to be associated with broadened orientation tuning, none of these models were also associated with activation data that match what would be expected based on published data on CS from chronic or FE schizophrenia.

In this section, and in Figures 3–8 (center and right panels or thin nonblue lines in Figure 8), the best fitting models for chronic schizophrenia and FES are described in detail. Models that were poor fits are described in supplemental Appendix B (Silverstein et al., 2017) and in Figures 9 and 10. To summarize what is presented in this section, the best fitting model for chronic schizophrenia involved the combination of reduced V1 lateral inhibition and changes in plasticity, whereas the best fitting model for FES involved a combination of reduced strength of input to LGN and V1, which was associated with overamplification of activity within V1.

### Effects of Simultaneous Changes in Inhibition and Other Parameters (Chronic Schizophrenia Model)

We evaluated the effects of reducing V1 lateral inhibition in combination with changes in the excitatory learning rate, the afferent learning rate, and the homeostatic adaptation rate. Combining 10% reduced V1 lateral inhibition with an increase in the V1 excitatory learning rate to 0.01 produced a close fit to published chronic schizophrenia findings. Activation was reduced at all contrast levels for low-contrast stimuli, although not to the extreme extent observed in some other models (see Model E data in Figures 9 and 10). However, activation at 80% contrast for the medium-SF stimulus was 9% higher than in the unmodified model, which is unrealistic. Alternative models, such as one in which the excitatory learning rate was set at 0.02, produced essentially the same effects. A closer fit to chronic schizophrenia data, however, was found in the model that combined 10% reduced V1 lateral inhibition with a threefold increase in the afferent learning rate. This generated reduced but realistic levels of activation at all contrasts at both SFs, except for the highest contrast/highest SF stimulus, for which the value was normal (see Figures 4C, 5B, and 8). Further reductions in inhibition with this model led to unacceptably low levels of activation, and further Increases in the afferent learning rate did not change the overall pattern. Interestingly, a nearly identical pattern of results was obtained from the combination of 10% reduced V1 inhibition and 90% decreased V1 homeostatic adaptation rate. These data suggest that increases in the rate at which V1 neurons update their activation weights regarding LGN input, or an increase in the duration for which V1 neurons can maintain excessive firing rates before returning to baseline levels via (slowed) homeostatic mechanisms, can account nicely for the CS findings observed in chronic (but not FES) patients.

These models also produced specific but different forms of OR tuning changes. Because the precise nature of the OR tuning changes in chronic schizophrenia are not well understood (e.g., a flatter peak of the tuning curve, a normal peak but excessive activity far from the peak, or both), both models that produced broadened tuning are described in an effort to generate hypotheses that can be tested in future studies with patients. In one such model, in which increased V1 excitatory learning was added to reduced V1 lateral inhibition, peak activation for selectivity was shifted to an orientation lower than 90°, and activation levels of neurons selective for orientations very different than the target were increased (see Figure 10E). Moreover, the strength of the connection weights for neurons at a distance from the target neuron were increased compared to that observed in the unmodified GCAL model (not shown). For the model in which the afferent learning rate was increased threefold, activation at neurons normally most selective for a vertically oriented stimulus was reduced, and a second peak emerged, with peak activation occurring for neurons selective for both lower and higher orientations (see Figure 7B). In this case, activation at neurons normally selective for orientations far from the target stimulus orientation was increased (as indicated by the high excess kurtosis value), but the connection weights between vertically selective and distant neurons were normal (not shown). Because this second model produced activation values regarding CS that were closest to data obtained with chronic schizophrenia patients, we consider it to be the best overall fit to the chronic schizophrenia data.

### Effects of Reduced Afferent Input to LGN and V1 (FES Model)

Models that specified reductions in both retinal and LGN output from between 10% and 50% were evaluated. Of these, the best fit to published data on CS in FES involved a 15% reduction in the strength of both variables (see Figure 8). This model produced increases in activation in response to the low-SF stimulus across a range of contrast levels, similar to what was observed by Shoshina et al. (2015), and normal levels of activation in response to the medium-SF stimulus (Figure 8; see also Figures 4, 5, and 6). At the same time, the overall OR preference map was still qualitatively similar to those observed in the unmodified GCAL model (see Figure 3C). The model with 10% reduced afferent strength produced the same overall pattern but with reduced effects for both low- and medium-SF stimuli. Models with greater than 15% reductions in afferent strength had the opposite effect: Activation values in response to the low-SF stimulus greatly exceeded the elevations in activation reported in published studies (e.g., elevations were up to 50% greater than unmodified GCAL values), and values in response to the medium-SF stimulus were lower (up to 7% lower than unmodified GCAL values) than in previous studies.

Adding the same parameter changes as used in the excitation/inhibition models (described in supplemental Appendix B (Silverstein et al., 2017) did not appreciably alter the pattern of activity observed when changing retinal and LGN afferent strengths alone. For example, slowing the V1 homeostatic learning rate by 90% in this model resulted in activation levels that were nearly identical to the model with only reduced afferent strengths. Adding an increased afferent learning rate (at the same 0.03 level used in the models described in supplemental Appendix B) to reduced afferent strengths generated activation levels that were also similar to the original model, but with higher activity values for the highest contrast low-SF stimulus. Increasing the excitatory learning rate to 0.01 had negligible effects on activation for the low-SF stimulus but increased activity to unrealistically higher than normal levels at mid-contrast levels for the medium-SF stimulus.

However, manipulation of these additional parameters did have effects on OR tuning. Increasing the V1 excitatory learning rate to 0.01 had the effect of shifting the peak of the tuning curve to a slightly lower orientation and raising the peak level of activation, while at the same time suppressing activity at other orientations, leading to steeper distribution tails. Increasing the afferent learning rate to 0.03 had the same effect as in other models, namely, a suppression of activity at the normally most selective neurons and emergence of a close to bimodal distribution (see Figure 10G). Decreasing the homeostatic learning rate had no effect on the shape of the OR tuning curves. These three models were exploratory in the sense that broadened OR tuning has not been demonstrated in FES (i.e., to our knowledge, there are no published reports on the issue). On the basis of the modeling findings, however, we hypothesize that if broadened OR tuning were found to characterize this population, it most likely would be due to increases in the V1 afferent learning rate.

We also examined the effects of increasing V1 lateral excitation and reducing V1 lateral inhibition strength in the model with reduced retinal and LGN efferent strength. These models did not produce good fits to published data. Increasing V1 lateral excitation strength dampened the effect of V1 activity relative to the model without this addition, such that an increase in activation, relative to the unmodified GCAL model, was found only at the highest contrast for the low-SF stimulus, which is the opposite of what would be expected in FES. Differences between this model and the unmodified GCAL model were more pronounced for the medium-SF stimulus, but here the combined model had excessively reduced activation, which also would not be expected. These effects were observed with as little as a 10% increase in V1 lateral excitation strength and were more pronounced when excitation was increased beyond this amount. When V1 lateral *inhibition* was reduced by 10% (the level used in models described earlier), effects on activation were negligible, and there were no changes to OR tuning curves.

Finally, we examined effects of either decreased retinal output or decreased LGN output alone. The model with 15% decreased retinal output alone generated activation levels that were very close to those of the unmodified GCAL model. The model with only 15% decreased LGN output produced slightly but consistently more activation than the model with only decreased retinal output for the low-SF stimulus but excessive activation at the lower contrast levels for the medium-SF stimulus. These data indicate that the combination of reduced retinal and LGN output leads to a closer fit to published FES data than either one alone.

### Single Best Fitting Model

For the sake of simplicity, all previously discussed models involved a deviation from the unmodified model after 10,000 training iterations. However, in the case of the two states of schizophrenia we attempted to model—unmedicated first episode and medicated chronic—there is clearly a temporal sequence. That is, first-episode psychosis is a relatively brief, acute phase, which is then followed by treatment-induced (at least) partial normalization of some functions. In most cases, this initial episode is followed by years of prolonged medication exposure, along with treatment- and illness progression–related changes, producing what appears as the chronic phase of the illness. Therefore a final set of models was run in which we (a) trained GCAL to either 10,000 or 20,000; (b) made the changes associated with the best fitting FES model (see description in caption for Figure 3C) and then ran the model for another 1,000 iterations to determine if, indeed, our hypothesized “acute” effects appear within this relatively short time scale; (c) normalized the retinal and LGN afferent strengths (but without resetting synaptic connection weights that were altered by the FES model), consistent with effects of antipsychotic medication; (d) introduced the parameter changes associated with the best fitting chronic schizophrenia model (see description in caption for Figure 3B); and (e) ran the model another 9,000 iterations to see if the “chronic” manipulations—when introduced to a visual system that was previously altered owing to an “acute” phase—generated the same alterations as when these changes were imposed on an otherwise normal system (e.g., the unmodified GCAL model at iteration 10,001). These models were run for both low- and medium-SF stimuli, with contrast fixed at 40%. With the unmodified model run first to 10,000 or 20,000, the activation in response to contrast and SF changes was nearly identical to that in the other nonsequential best fitting chronic and FES models described previously, differing at most by a value of .002 units in activation. OR tuning data were also similar to these other models. These data confirm both that the changes we observed in our best fitting FES model occur acutely and that the changes observed in the chronic model can occur after acute effects have altered the function of the system.

## DISCUSSION

The primary goals of the computational modeling efforts described here were to (a) determine whether changes to a limited number of parameters could account for increased CS in unmedicated FES versus decreased CS in medicated chronic schizophrenia and (b) determine whether the same manipulations that led to decreased CS in chronic schizophrenia would also lead to broadened OR tuning. An excellent fit was observed between published findings in chronic schizophrenia and a model incorporating a combination of 10% reduced V1 inhibition and a tripling of the Hebbian learning rate for afferent connections from LGN to V1. This led to both reduced CS for low-SF stimuli and broadened OR tuning. For FES, the model that most closely simulated published findings on increased CS involved a reduction of afferent signal strength from both the retina and LGN. This model led to increased V1 activation in response to the low-SF stimulus but normal activation in response to the medium-SF stimulus. Of note, this model did not produce broadened OR tuning (which has not been reported in FES). However, adding an increase in the V1 afferent learning rate to the model broadened OR tuning in the same way as occurred in the chronic schizophrenia model, *while preserving the increased and normal levels of activity in response to low- and medium-SF stimuli, respectively*. Broadened OR tuning was also seen in the FES model with the addition of a large (90%) decrease in the rate at which homeostasis in V1 firing rates is reestablished. Therefore, should broadened orientation tuning be demonstrated in FES in the future, these mechanisms should be explored as potential causes.

### Consistency With Prior Findings

Our data are consistent with the idea that while occipital lobe GABA levels are reduced in schizophrenia (Cadenhead et al., 2013; Kelemen et al., 2013; Yoon et al., 2010), this may not be as prominent at the first episode of psychosis as it is later in the condition, owing to aging effects. For example, cortical GABA levels (Marsman et al., 2014), the ratio of GABAergic to non-GABAergic neurons (Hua, Kao, Sun, Li, & Zhou, 2008), CS (Z. Wang et al., 2014), and OR tuning (Betts, Sekuler, & Bennett, 2007; Hua et al., 2006; Yu, Wang, Li, Zhou, & Leventhal, 2006) all decline with normal aging, suggesting that a reduction in GABAergic function and/or number of inhibitory neurons in visual cortex may play a role in the accelerated cognitive aging that has been hypothesized to occur in schizophrenia (Kirkpatrick, Messias, Harvey, Fernandez-Egea, & Bowie, 2008). These data also support the hypothesis that reduced CS and broadened OR tuning share common mechanisms and that these mechanisms do not become operative in people with schizophrenia until some time after the first episode of psychosis.

Beyond aging effects, several other lines of evidence support the hypothesis that reduced GABA concentration in visual cortex and therefore reduced inhibition are involved in reduced CS and broadened OR tuning in chronic schizophrenia. First, it has been shown in cat and monkey studies that reductions in V1 inhibition are related to reductions in CS (Zhou, Shi, Peng, Hua, & Hua, 2011). Second, in primates, reduced CS secondary to reduced inhibition (Z. Wang et al., 2014) and OR selectivity are both effects that emerge at the level of V1 (Isaacson & Scanziani, 2011), and so they cannot be implemented by retinal and LGN mechanisms alone, in contrast to the changes characteristic of our best fitting FES model. Third, inhibition has long been thought to play a role in sharpening tuning of cortical neurons (Isaacson & Scanziani, 2011). Fourth, pharmacological blockade of GABA_*A*_ receptors reduces stimulus selectivity in multiple sensory cortices, including for OR selectivity in visual cortex (Katzner et al., 2011). Fifth, in healthy humans, individual performance on an OR discrimination task was significantly correlated with resting V1 GABA concentration (Edden, Muthukumaraswamy, Freeman, & Singh, 2009). Sixth, prolonged GABA reduction can lead to reactivation of forms of experience-dependent plasticity that are normally not possible after early development. For example, Harauzov et al. (2010) observed that a reduction of GABAergic activity in rat visual cortex led to a reemergence of ocular dominance plasticity. This raises the possibility that a chronic reduction in GABAergic transmission could lead to a reactivation of plasticity in OR maps, a process that is normally completed very early in life. This is precisely the alteration that was modeled by our changes in the V1 afferent learning rate in combination with reduced V1 lateral inhibition. This combination of modifications increased the influence of recent activity over the long-term correlations stored in the model, thereby progressively dismantling the sharpening of tuning that was achieved with normal development and leading to broadening of OR tuning curves.

An interesting aspect of the chronic schizophrenia models we generated is that reduced V1 lateral inhibition led to what may appear to be a paradoxical reduction in activation in most cases. One way to understand this is to consider that reductions in inhibition will have the effect of increasing excitation, which will then prompt a broad inhibitory response. In the GCAL class of models, this is possible because activity settles over 20 steps, with most of these occurring after input to the cortex. Also, the inhibition we manipulated was lateral inhibition, not total inhibition. For example, in the best fitting model for the chronic schizophrenia data, homeostatic mechanisms remained in place to keep overall V1 activation at the target level. Regardless of the mechanism, however, the evidence reviewed indicates that reductions in V1 inhibition are related to both reduced CS and broadened OR tuning. We therefore believe that our models are behaving in biologically realistic ways, even if the specific mechanisms that implement them are not fully known at present.

Our FES model suggests the hypothesis that reduced retinal and LGN signaling can lead to increased CS. Although this hypothesis has not yet been tested in patients, it is supported by several lines of evidence. For example, 4 hours of exposure to a low-contrast visual environment (via wearing special lenses that reduce contrast input) produced significant changes, such as improvement in contrast-discrimination sensitivity as measured psychophysically and an increase in the gain of fMRI contrast response functions in V1 and V2 (Kwon, Legge, Fang, Cheong, & He, 2009). Also consistent with our FES model are results of a mouse study in which a reduction in visual input (via retinal lesions) led to a long-lasting reduction in the number of inhibitory cell dendritic spines in visual cortex (Keck et al., 2011). Because these spines form glutamatergic synapses, these data suggest that structural changes in inhibitory neurons that arise after reduced visual input can lead to increases in excitatory activity and that this is a basic mechanism involved in adaptation following sensory deprivation. Although it is not known whether similar effects arise from reduced retinal signaling, this is a testable hypothesis. It is also consistent with current findings that in response to a reduction in sensory input, there is recovery of excitatory activity and a reduction in inhibitory activity (Barnes et al., 2015). Applied to schizophrenia, this work is consistent with literature suggesting that some aspects of psychosis resemble effects of sensory deprivation (Corlett, Frith, & Fletcher, 2009; Rosenzweig, 1959). It also suggests a link between reduced retinal signaling and the emergence of visual hallucinations, via reduced V1 inhibition. This relationship has not been adequately explored, although it is consistent with the findings of Balogh et al. (2008), who reported that reduced photoreceptor signaling (ERG a-wave amplitude) was associated with more severe positive symptoms in acutely ill patients.

The changes in inhibition and excitation discussed herein may interact with normal gain control mechanisms to further amplify signal strength in visual cortex. For example, contrast gain control in LGN On and Off channels increases the responses to low-contrast stimuli more than it increases responses to high-contrast stimuli (Olman, Ugurbil, Schrater, & Kersten, 2004). It has also been shown that V1 responses, as measured by the fMRI BOLD response, to low-SF stimuli are weaker than those for medium-SF stimuli (Goodyear et al., 2000). Perhaps, then, it is primarily for low-contrast stimuli that reduced retinal and LGN signaling (which our models indicate are found in FES) lead to abnormally low input to V1, thereby leading to compensatory changes that amplify V1 activity above normal levels. This may be magnified further by tighter coupling between thalamic and cortical sensory processing regions in schizophrenia compared to healthy controls (Anticevic et al., 2014), in the sense of increasing the total amount of weak V1 stimulation, for which V1 overcompensates by increasing its activity. All of this is consistent with what has been observed psychophysically in CS tasks among FES patients. It also fits with the many clinical reports of increased intensity of perception in FES prior to treatment and the reduction in these experiences after treatment (Bunney et al., 1999; Keri, Kiss, Kelemen, Benedek, & Janka, 2005; Phillipson & Harris, 1985).

Our modeling data are also consistent with findings that at the first episode of psychosis, there is excessive cortical activation, but that this does not persist into the chronic phase of illness owing to medication use, aspects of illness progression (e.g., reduction in inflammation), aging, or some combination of these or other factors (Anticevic et al., 2015; Rivolta et al., 2014; Rivolta et al., 2015; Silverstein et al., 2012). This change over time fits with ketamine models of schizophrenia, in which NMDA receptor blockade and excess glutamate transmission produce effects that more closely resemble features found in acute psychosis rather than in chronically ill patients (Anticevic et al., 2015; Frohlich & Van Horn, 2014; Phillips & Silverstein, 2003). These data also fit with findings that brain glutamate levels are increased in the early stages of the disorder, especially in unmedicated patients (Poels et al., 2014), followed by age-related reductions that are more marked in people with schizophrenia than in healthy controls, such that glutamate levels fall below those of controls by around age 25 years, typically after a few years of illness (Marsman et al., 2014; Marsman et al., 2013). Note that an alternate best fitting model to the FES data, with the assumption that OR tuning is broadened at this stage of illness, involved an increase in the afferent learning rate for LGN synapses onto V1, a change that is consistent with increased glutamatergic activity (Corlett et al., 2011).

Finally, changes in DA functioning may be a mechanism underlying effects in the FES model. This suggestion comes from two sources of evidence. One is that thalamo-cortical connectivity is modulated by DA activity in the striatum (Alexander & Crutcher, 1990) and that striatal DA levels are positively correlated with ratings of subjective visibility (Van Opstal et al., 2014). Because striatal DA activity is elevated in schizophrenia (Kegeles et al., 2010), it is possible that this increases the gain of (weakened) visual input to V1, thereby contributing to compensatory adaptation effects in V1 that may overshoot target activity levels in un treated FES. Also, as noted earlier, DA is known to suppress activity in rod photoreceptors (Witkovsky, 2004), and so findings of excessively low ERG amplitudes in acutely psychotic patients (Silverstein & Rosen, 2015) are consistent with *increased* retinal DA, as are findings of a significant correlation between reduced ERG amplitudes and more severe positive symptoms (Balogh et al., 2008). At the other extreme, and potentially relevant to patients with chronic schizophrenia being treated with DA antagonists, is reduced retinal DA, as observed in Parkinson’s disease; this has been associated with reduced CS (Brandies & Yehuda, 2008). While retinal DA levels have not been described in schizophrenia, several studies (reviewed in Brandies & Yehuda, 2008) have indicated that antipsychotic medications block D1 and D2 receptors in the retina, although different medications may block the two receptor subtypes to different degrees. Thus a similarity in CS between chronic schizophrenia and Parkinson’s disease patients, owing to decreased retinal DA, is a reasonable hypothesis to test in future studies. This hypothesis is also consistent with previously reported effects of medication treatment on partially normalizing ERG amplitudes (Balogh et al., 2008).

### Testable Hypotheses Suggested by the Models

Our data suggest several hypotheses for future experimental studies in patients. For example, the chronic model suggests that decreased CS and broadened OR tuning should be strongly correlated in chronic schizophrenia patients (a question that, to our knowledge, has not yet been examined), which, in turn, should be related to an interaction between reduced inhibition and a faster than normal updating (i.e., reduced stability) of afferent weights between LGN and V1 neurons. This model also predicts that broadened OR tuning will be most pronounced at higher SFs (see Figure 5) and that it will be associated with a reduction in peak sensitivity for orientations for which V1 neurons are normally tuned (see Figure 7), another hypothesis that has not yet been tested. In addition, as noted earlier, if broadened OR tuning is observed in FES patients in future studies, increased V1 afferent learning and a slowing in homeostatic mechanisms should be investigated as competing potential mechanisms of this effect.

The FES models suggest that the pronounced reduction in ERG amplitudes observed in newly treated patients (Balogh et al., 2008) should be related to the especially pronounced reduction in VEP amplitudes also found in unmedicated patients (Connolly, Gruzelier, Manchanda, & Hirsch, 1983; Jutai, Gruzelier, Connolly, Manchanda, & Hirsch, 1984; Shagass & Roemer, 1991; Shagass et al., 1982; Straumanis et al., 1982) and to the higher CS found in untreated FES (Cadenhead et al., 2013; Kelemen et al., 2013; Kiss et al., 2010; Shoshina et al., 2015) but not necessarily to any OR tuning changes. Additionally, because the FES model simulated psychophysical findings without resorting to a change in inhibition, it suggests that the perceptual changes observed in this group primarily reflect dopaminergic and glutamatergic changes (see following discussion), whereas changes in GABAergic tone (at least in V1) arise later in the course of illness, as discussed previously.

### Limitations

A number of important limitations of this work must be mentioned. One is that a model is only a model. Just because a model generates a good fit to human or animal data does not mean that it captures the real state of nature. There are potentially many ways in which a dataset can be mathematically modeled, and this increases with the number of parameters available for manipulation. Keeping this in mind, we emphasize that the purpose of the modeling efforts presented here is not to provide a definitive solution to a problem but rather to constrain and generate useful testable hypotheses that can help advance our understanding of schizophrenia. Beyond this, however, there are specific limitations to the work we presented, and these are discussed in the following paragraphs.

As described in Methods, the GCAL model LGN sheets do not represent only the LGN but also the bipolar and retinal ganglion cell layers. This is potentially relevant to our results because we did not observe an LGN effect that might have been expected, namely, reduced gain control in the chronic schizophrenia model. On the other hand, reducing LGN afferent strength, which was an important manipulation for the chronic schizophrenia model, is consistent with reduced gain control. This raises the question of whether a model with a more restricted and specialized LGN layer might have produced different results. Such a model, which goes beyond GCAL in several ways, including by incorporating separate populations of excit atory and inhibitory cells, different sheets for retinal ganglion and LGN cell types, and multiple cortical layers, has been shown to model many visual effects successfully (Bednar, 2014b). It is possible that this extended GCAL model, or others that have yet to be developed, might help resolve precisely where and how gain control mechanisms are disordered in schizophrenia. It is also possible, of course, that LGN gain control really is intact in schizophrenia and that what has been interpreted as reduced gain control are actually effects of reduced cortical (V1 or higher) inhibition or even possibly abnormalities in post-photoreceptor retinal activity (e.g., in bipolar or ganglion cells or their circuitry). This distinction needs further testing, especially given that our best fitting chronic schizophrenia models included reduced V1 lateral inhibition and that multiple research findings suggest that this is an issue in schizophrenia.

A third limitation is that while GCAL model neurons are broadly tuned and are sensitive to a range of SFs, their *peak* sensitivity varies very little because GCAL receptive field sizes are identical in all cases (based on the same difference of Gaussians),^3^ whereas in human brains, a range of receptive field sizes is found. The SF to which GCAL is most sensitive is approximately halfway between the SFs of the two stimuli we used to test the model. Because of this, (a) the generalizability of our model data to systems in which multiple receptive field sizes exist is unknown and (b) the generalizability of our model data to neurons responding to stimuli to which they are optimally tuned is unknown. On the other hand, because V1 neurons will respond to SFs other than the one to which they are optimally tuned (with decreasing levels of responsiveness with increased distance from the orientation associated with peak sensitivity, as shown in Figures 7 and 10), our data may nevertheless be accurate in depicting the *relative* differences in activation between lower and higher SF stimuli. We say “relative” here because if we had developed a (very computationally expensive) model that included a large range of SF preferences, we would assume that peak activation and most likely total activation levels would have been higher than the ones we observed (owing to many more neurons reaching their peak activation levels at least some of the time). However, we believe that the relative distinctions are more important than the actual values for our purposes in this article. This is because it was not our intention to model actual levels of neuronal activation but rather to model differences in the size (and direction) of changes in the two patient groups relative to psychiatrically healthy subjects. Also, despite the limitation on generalizability arising from our use of only a single receptive field size, it must be noted that low-contrast and low-SF stimuli produce lower activation levels than higher SF stimuli (Boynton et al., 1999; Goodyear et al., 2000), presumably owing to less light reaching the retina and less center-surround antagonism with stimuli that cover a larger region of space, respectively. Therefore lowering retinal and LGN efferent strength in the FES model would be expected to lead to weaker V1 input with low-contrast stimuli than with higher contrast stimuli, as well as to corresponding V1 amplification, regardless of SF. That we observed this should not be surprising given the strong sensitivity of GCAL to changes in contrast (Stevens et al., 2013). Similarly, we would expect that reduced inhibition in V1 would reduce CS regardless of SF (at least at low contrast levels), which is what we observed in our chronic schizophrenia model.

A fourth potential limitation is that our model assumed that V1 function in schizophrenia can be simulated by normal development of V1 structure and function up to the point where perturbations of retinal, LGN, and/or V1 function were introduced. This may be an overly conservative assumption, given evidence of abnormal brain function as early as infancy in people who go on to develop schizophrenia (Fish & Kendler, 2005), an increased rate of conditions that cause abnormal visual development (e.g., strabismus) in children at risk for schizophrenia (Schiffman et al., 2006; Schubert, Henriksson, & McNeil, 2005), and reductions in ERG waveforms in offspring of mothers with schizophrenia (Hebert et al., 2010). On the other hand, there is a lack of evidence specifically regarding CS and orientation tuning in the premorbid and prodromal periods, and given this, we thought it would be best to explore models that develop normally until late adolescence/early adulthood. Our assumption that GCAL at 10,000 iterations is roughly equivalent to late adolescence or adulthood is based on prior demonstrations that at this stage, robust and stable orientation maps are evident that are associated with many adultlike perceptual effects, even though they still continue to be refined with additional iterations (Miikkulainen et al., 2005; Stevens et al., 2013).

A fifth limitation of the study involves our aim to develop models only of untreated FES and chronic medicated schizophrenia. This necessarily would lead to an incomplete picture of schizophrenia, as it would not represent medicated FES patients or unmedicated chronically ill patients, thereby limiting our ability to disentangle developmental, illness, and treatment effects. On the other hand, modeling these two groups allows for potential reconciliation of opposite findings regarding CS, which, to our knowledge, has not previously been attempted, and so this comparison can lead to useful insights into differences between the two populations, even if the precise influences of age and medication cannot be disentangled at this point. On a more practical level, though, nearly all existing data on CS come from these populations: There is little published CS data on unmedicated chronic patients, and all data on OR tuning come from medicated chronic patients. Therefore there is very little basis, at this time, for modeling these functions in these other populations.

An additional limitation related to our choice of populations to model is that while the CS findings we used as the basis for our FES model predictions indeed came from studies of unmedicated FES patients, the ERG data we used as part of the basis of the FES model were from studies of acutely psychotic later-episode patients whose performance was shown to change after multiple weeks of medication (Balogh et al., 2008). Of course, our FES model assumes and predicts that unmedicated FES patients would demonstrate reduced ERG waveform amplitudes. If this were to be disconfirmed, it would invalidate the model. On the other hand, it is also possible that if our model is valid for first-episode patients, it might also be valid for later-episode unmedicated patients experiencing acute psychosis and thus more related to the severity of psychosis than to a specific developmental point in the course of illness. However, there is reason to believe that illness chronicity does matter. For example, O’Donnell et al. (2006) demonstrated that both medicated and unmedicated *chronic* schizophrenia patients were characterized by reduced CS, suggesting that with illness progression, a decline in this function is evident, regardless of medication status. On the other hand, Chen et al. (2003) found increased CS in unmedicated chronic patients, although only six such patients were included in that study. Related to OR tuning, Rokem et al. (2011) observed that medication dose accounted for only 1% of the variance in the orientation tuning data of schizophrenia patients. Our hope is that our findings will motivate studies of retinal and LGN function in relation to V1 activation and psychophysical task performance (which have not been conducted), across developmental stages of schizophrenia, in both medicated and unmedicated patients, so as to clarify the relative contributions of psychosis, chronicity, and medication effects.

A final limitation regarding heterogeneity is that even within patients at the same stage of illness and with the same medication status, there is considerable variability in the nature and severity of symptoms and of perceptual and cognitive functioning. Therefore, at best, models such as the ones we present here can be seen as relevant to groups of patients but not necessarily to all patients within a group.

A sixth limitation of the study involves our assumption that V1 GABA levels are reduced in chronic schizophrenia and that this is related to reduced CS and broadened OR tuning. Although much evidence is consistent with this hypothesis (reviewed previously), only two studies have actually investigated the correlation between V1 GABA levels and CS: One did not find a significant relationship (Kelemen et al., 2013), and the other observed a relatively high value of *r*, in the predicted direction (− 0.40), which was at the trend level of significance (Rokem et al., 2011). On the other hand, the lack of a stronger linear relationship between V1 GABA concentration and CS is consistent with our chronic schizophrenia model result that reduced inhibition *alone* did not account for findings on CS or broadened OR tuning. However, in interaction with other factors, such as an increased afferent learning rate, the role of reduced inhibition was important. Therefore a more nuanced understanding of the role of inhibition in low-level visual processing deficits in chronic schizophrenia appears to be necessary, as does a better understanding of GABAergic abnormalities, and perhaps more precise measurement of them, because at present, GABA levels can be measured only indirectly (Taylor & Tso, 2015).

Seventh, our models did not incorporate changes in retinal and V1 structure that have been observed in schizophrenia. For example, multiple studies have reported retinal nerve fiber layer (i.e., ganglion cell axon) and/or macular thinning in schizophrenia (reviewed in Silverstein & Rosen, 2015). Studies have also demonstrated V1 atrophy (loss of gray and white matter) in people with schizophrenia, especially in those with a chronic course and poor outcomes (Mitelman & Buchsbaum, 2007; Selemon, Rajkowska, & Goldman-Rakic, 1995; Van Rheenen et al., in press). At present, the relationships between these anatomical findings and changes in visual perception are not known. However, if such relationships do exist, then future models would need to incorporate the relevant parameter changes. We did explore models in which neuronal density in the retina was reduced, and this alone did not generate data consistent with chronic or first-episode patients. However, this model began at iteration 0 with reduced retinal density because there is no feasible way in the current version of Topographica to modify either retinal or cortical density in GCAL once visual cortex development has begun, and so a realistic developmental model of schizophrenia is currently not possible with changes in these parameters.

Finally, it is important to note that the effects we demonstrated were modeled without recourse to top-down processes. While this demonstrates that effects such as reduced CS and broadened OR tuning can result from changes in feedforward and lateral signaling only, it of course does not mean that this is the situation in nature. For example, attention can increase response gain in visual cortex (Thiele, Pooresmaeili, Delicato, Herrero, & Roelfsema, 2009), and because attention is reduced in many people with schizophrenia, there is always the possibility that reduced CS in chronic schizophrenia is due in part to attentional effects. On the other hand, attentional effects do not operate at the level of retinal responses (Hackley, Woldorff, & Hillyard, 1990), and they are not consistent with increases in CS for low-SF stimuli in FES. However, there are also forms of low-level top-down feedback that have yet to be explored in experimental or computational studies of schizophrenia. These include inhibitory feedback from the perigeniculate sector of the thalamic reticular nucleus to the LGN (Vaingankar, Soto-Sanchez, Wang, Sommer, & Hirsch, 2012) and intraretinal feedback such as that from horizontal cells to photoreceptors (Thoreson, Babai, & Bartoletti, 2008). Therefore continued clarification of the extent of bottom-up and lateral processing contributions to low-level visual disturbances in schizophrenia, in addition to the role of top-down effects at various levels, promises to further inform our understanding of the disorder.

Despite these limitations, we believe it is noteworthy that computational models of low-level visual processing can demonstrate relationships between phenomena that have previously been considered only separately. For untreated FES, this includes reduced retinal and LGN signaling and excessive CS to low, but not higher, SF information. For chronic, medicated schizophrenia, this includes reduced V1 inhibition, reduced CS, and broadened OR tuning. The computational models we present here can thus be considered to function as frameworks that provide order and parsimony to existing data and that lay the groundwork for future hypothesis testing in patient samples. If these models-as-theories stimulate experimental work that moves the field closer to a more comprehensive understanding of schizophrenia, then they can be considered useful, despite the assumptions and oversimplifications inherent to modeling approaches in general, and indeed to most research where complex phenomena must be operationalized in simpler forms.

## AUTHOR CONTRIBUTIONS

Steven M. Silverstein: Conceptualization: Lead; Data curation: Lead; Formal analysis: Lead; Investigation: Lead; Methodology: Equal; Project administration: Lead; Writing – original draft: Lead; Writing – review & editing: Lead Docia Demmin: Conceptualization: Supporting; Project administration: Supporting; Software: Supporting; Writing – original draft: Supporting; Writing – review & editing: Supporting James Bednar: Methodology: Equal; Resources: Equal; Software: Equal; Writing – review & editing: Equal

## ACKNOWLEDGMENTS

The authors thank Pamela Butler, Molly Erickson, and Judy Thompson for their constructive criticism on earlier drafts of this article. We also thank Jean-Luc Stevens and Philipp Rudiger for their excellent technical support regarding GCAL and Topographica and Attila Farkas for critical assistance with Linux and Python.

## Notes

^1^ We also ran models that were tested (posttraining) with horizontal and oblique (45°) gratings, but the results did not differ from those obtained with vertical gratings, and so only the vertical grating data are reported here.^2^ We also calculated standard deviation and kurtosis values for each stimulus for each model. However, because the former values did not appear to be meaningfully related to the model manipulations, these are not reported here. Kurtosis values are reported where relevant. In addition, we calculated maximum activation values. Because these were highly correlated with mean activation values, they are only reported in illustrative cases when describing between-condition differences in activation maps.^3^ Although a single receptive field size was used, there is still *some* variation in peak sensitivity because of differences in the strength of orientation tuning across neurons: Neurons poorly tuned for orientation have circular receptive fields, whereas neurons strongly tuned for orientation have narrower On and Off zones in their receptive fields. This is true for both real visual cortex neurons across many species and for GCAL neurons.
